# Study of Melamine-Formaldehyde/Phase Change Material Microcapsules for the Preparation of Polymer Films by Extrusion

**DOI:** 10.3390/membranes12030266

**Published:** 2022-02-25

**Authors:** Sara García-Viñuales, César Rubio, Lidia Martínez-Izquierdo, Beatriz Zornoza, Elena Piera, Miguel Ángel Caballero, Carlos Téllez

**Affiliations:** 1 Instituto de Nanociencia y Materiales de Aragón (INMA), CSIC-Universidad de Zaragoza, 50009 Zaragoza, Spain; sgarvi@unizar.es (S.G.-V.); cesar.rubio@unizar.es (C.R.); limartinez@unizar.es (L.M.-I.); 2 Chemical and Environmental Engineering Department, Universidad de Zaragoza, 50018 Zaragoza, Spain; 3 Research and Development Department, Nurel S.A., Ctra. Barcelona km 329, 50016 Zaragoza, Spain; epiera@samca.com (E.P.); acaballero@samca.com (M.Á.C.)

**Keywords:** n-Eicosane-melamine formaldehyde microcapsules, polyamide 6 films, phase change material, heat storage

## Abstract

n-Eicosane-melamine formaldehyde microcapsules of an average size of 1.1 μm and latent heat of fusion of 146.2 ± 5.3 J/g have been prepared. They have been characterized by scanning electron microscopy, FTIR spectroscopy, calorimetric techniques, and thermogravimetric analyses. Under processing conditions, the microcapsules apparently preserved their properties, also maintaining their n-eicosane loading and heat storage capacity under washing conditions (water with detergent at 60 °C). The microcapsules synthesis has been scaled up for the fabrication of functional films by extrusion. For that, polymer films containing 10 wt.% of microcapsules were prepared at a pilot plant level. In those films, even though a fraction of the n-eicosane loading was lost during the extrusion process, the microcapsules showed good compatibility within the polyamide. The percentage of PCM in the polyamide 6 films was estimated by TGA, verifying also the heat storage capacity predicted by DSC (2.6 ± 0.7 J/g).

## 1. Introduction

In recent years, the growth of the textile industry has been significantly stimulated by the development of functional polymeric materials using microcapsules that cover various applications (fashion, sports, protective clothing, cosmetics or health care, among others) [1,2]. Different polymeric membrane/film/fiber preparation methods have been extensively used according to the preferred final material morphology, i.e., phase inversion [3], controlled stretching [3], electrodeposition [4], interfacial polymerization [5] and extrusion [6,7].

In a previous work, polyamide (PA) fibers were fabricated with porous inorganic microcapsules (MCs) that were resistant to the extrusion temperature. In these MCs various additives were encapsulated and incorporated inside the yarn. With this strategy, it was possible to obtain an improvement in the strength and durability of the product [7]. However, the compatibility between the polymer and the inorganic material was not good as expected, and there is still room for improvement with the use of polymeric materials as encapsulating materials. It is also worth mentioning that the inorganic capsule formation process involves two different stages: first, the preparation of the inorganic microcapsule, and second, the additive encapsulation. In this sense, the fabrication of polymeric MCs would enhance the process efficiency, since it is possible to prepare the microcapsule and encapsulate the additive at the same time. Nevertheless, there is still limitation of temperature since the polymer must withstand the extrusion conditions (260 °C in the case of PA). In the open literature, there is a great variety of thermo-resistant polymers with good mechanical stability [8] that degrades above the extrusion temperature [9,10]. Among them, melamine-formaldehyde (MF) has been selected for this research. MF has already been used for the encapsulation of additives such as oils [11], perfumes [12,13] or phase change materials (PCMs) [14,15] due several advantages (i.e., low price, easy-to-control preparation, high compatibility and good thermal stability). As encapsulating agents, different PCMs have already been incorporated into MF microcapsules. Some examples are n-octadecane [8,16], paraffin wax [17], 1-dodecanol [18], and n-eicosane [9,19]. PCMs are materials capable of absorbing and releasing large amounts of energy in the form of latent heat (ΔH) during the phase transition between liquid–solid at a given temperature limit [20,21]. They have many possible applications, ranging from the stabilization of temperature to heat or cold storage [22].

Therefore, the encapsulation of PCMs and their introduction into polymers would allow for the obtaining of fabrics that absorb or provide heat, substantially improving the user’s feeling of comfort. This could be an interesting application for the sport’s industry. To choose an appropriate PCM, it is crucial to understand the changes that occur in that material during its utilization [23]. For example, an ideal PCM for its use as regulating agent for body temperature would be n-eicosane. The solid–liquid phase transition of n-eicosane occurs at 37 °C which is usually the human body temperature. In this case, when the body temperature exceeds 37 °C, the compound will absorb heat (endothermic process), while if temperature decreases, the paraffin will crystallize (exothermic process) releasing heat. Since the phase change is reversible, the absorption/release of heat will occur whenever the corresponding temperature change occurs, which will allow a garment to provide thermal comfort. This will help our body to better adapt to sudden changes of temperature.

Mohaddes et al. [9] developed MF/n-eicosane MCs with diameters around 2.1 µm and incorporated them into polyester viscose fabric pieces with a coat-dry-cure method. Nevertheless, the authors fabricated them using surface coating, which is a less durable method than their introduction into the polymer during the extrusion process.

The purpose of this work is to study the preparation of polymeric MCs with a PCM that can be used during extrusion processes. Specifically, MF/n-eicosane MCs were synthesized. Compared to inorganic materials, already proved in previous work developed by our group [6,7], these polymeric MCs will allow *(i)* a greater MCs loading, *(ii)* a reduction in the number of stages for its production, *(iii)* an improvement of the encapsulation efficiency, since the majority of the additive remains in the microcapsule, and (*iv*) better compatibility between the additive and the MC. To analyze the behavior of these MCs during the extrusion process, as well as approaching the preparation of textile fibers, polyamide 6 (PA6) and low-density polyethylene (LDPE) films with embedding PCM n-eicosane-loaded MF MCs have been prepared at a large scale. Moreover, the heat storage capacity of the MCs under clothing washing conditions has also been studied.

## 2. Materials and Methods

### 2.1. Materials

Monomers used were melamine (99%, Acros Organics, Geel (Belgium)) and formaldehyde (37%, ACS reagent, stab. 10–15% methanol, Acros Organics). n-eicosane (99%, Acros Organics) was used as the encapsulated material and sodium dodecylbenzenesulfonate (SDBS, technical grade, Sigma Aldrich, Darmstadt (Germany)) as surfactant in the emulsion process. To regulate the pH during the synthesis, sodium hydroxide (pellets, Scharlab, Barcelona (Spain)) and acetic acid (glacial, 99%, Alpha Aesar, Kandel (Germany)) were used.

### 2.2. Synthesis of Microcapsules (MCs)

The synthesis of MF/n-eicosane MCs consisted of three stages: emulsion, pre-polymerization and polymerization [12]. According to [24,25] the condensation reaction and the resulting structure of the MF MCs are strongly affected by the reaction conditions: molar ratios of reactants, pH and reaction temperature profiles during MCs formation. In the emulsion process, aqueous and organic phases were mixed in the presence of surfactant. The size of the MCs depends in part on the size of the drops in the emulsion, which is mainly related to the mechanical agitation speed and the surfactant used. During the pre-polymerization, melamine and formaldehyde reacted in a 1:3 stoichiometric ratio, forming the pre-polymer that was subsequently added to the emulsion [26]. At this point the polymerization stage began, with the polymer forming around the surfactant trapping the paraffin inside. As aforementioned, the pH also plays a crucial role in the condensation reactions of melamine and formaldehyde. If the pH is relatively low (7–8), methylene bridges dominate, whereas at higher pH values (above 9), ether bridges are favored [26]. Therefore, this parameter must be properly regulated.

**Figure 1:**
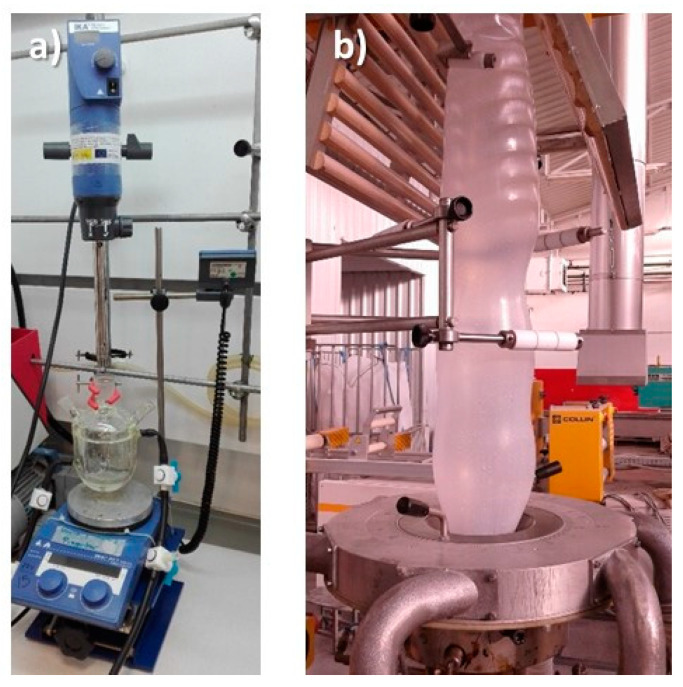
(**a**) Experimental set-up for the preparation of microcapsules; (**b**) extrusion process of a LDPE film with MF/n-eicosane microcapsules.

In this work, the MCs were prepared according to the following literature references, [9,12,26]. The emulsion was carried out in a three-necked round bottom jacketed flask, where 50 mL of an aqueous solution of SDBS (2% *w*/*v*) and 7.5 g of n-eicosane were introduced. The mixture was then emulsified at 8000 rpm using a T 25 digital ULTRA-TURRAX^®^ (IKA; Staufen, Germany) for 2 h at 70 °C. Pre-polymerization was prepared with 1.2 g of formaldehyde (37%) dissolved in 50 mL of water. An amount of 0.6 g of melamine was then added and basified to pH 10 by adding NaOH (10% *w*/*v*). Melamine is not soluble in water, so the pre-polymer was fully formed when the solution was completely clear. In the final polymerization stage, the pre-polymer was added dropwise to the emulsion and for the proper formation, according to the literature [9], pH was adjusted to 4 by adding acetic acid (10% *w*/*v*). The mixture was kept mechanically stirring for 2 h at 70 °C, forming a white paste. This paste was filtered and washed with water at 70 °C. Finally, the sample was dried overnight at room temperature obtaining a white powder. Figure 1a shows the experimental set-up for this synthesis.

For scaling-up, the synthesis process described above was carried out in a 1 L flask for the emulsion stage and a 250 mL flask for the pre-polymerization stage. The amount of each reagent is listed in Table 1. The prepared samples were named as MCX, where X indicates the amount of reagents relative to the unscaled sample. Thus, the unscaled sample would be the MC1 sample and the MC12 sample the one in which reactants have been multiplied by 12. Scaling-up at laboratory was required for further preparation of PA6 and LDPE films containing the MCs.

### 2.3. Manufacture of Polyamide and Low-Density Polyethylene Films Modified with Microcapsules

For this process, extrusion tests of PA6 and LDPE composites with MF/n-eicosane MCs were performed on a single screw machine. Figure 1b shows how the LDPE film with the embedded MCs was obtained.

According to our previous experience in the preparation of composite PA6 films with inorganic microcapsules (2 wt.% of disaggregated titanosilicate JDF-L1) [6], in this work, the extrusion tests were carried out at higher loadings: between 9 and 17 wt.%. Moreover, the extrusion temperature was varied between 235 °C and 256 °C. To obtain these percentages, 36–68 g of MCs and 364–332 g of pure polymer were added. Tests on LDPE using the equipment in Figure 1 allowed working at a temperature of 133 °C, much lower if compared to the one needed for PA6 films.

### 2.4. Characterization

The morphology of the MCs and films was studied using a scanning electron microscope (SEM) INSPECT F50 (FEI company, Eindhoven, The Netherlands). SEM experiments were performed at 2–15 kV after pre-coating the samples with platinum.

The shape of the MCs was determined using the sphericity factor (S.F.) [27,28], calculated from the following formula (Equation (1)):

(1) $$S.F.=\frac{d_{\max}-d_{\min}}{d_{\max}+d_{\min}}$$

where d_max_ is the maximum diameter and d_min_ is the minimum diameter perpendicular to d_max_ of the same MC.

Thermal properties of MF/ n-eicosane MCs and the films modified with those MCs were studied by differential scanning calorimetry (Mettler Toledo DSC822^e^, Columbus, OH, USA) and thermogravimetric analysis (Mettler Toledo TGA/SDTA 851^e^, Columbus, OH, USA). Analyses with both techniques were carried out under a nitrogen atmosphere. The heating ramp for TGA was 10 °C/min up to a temperature of 750 °C. In DSC, two heating ramps were used, with 1 °C/min and 10 °C/min, being the one at 1 °C/min more precise to determine the melting temperature. Different temperature programs were used depending on the objectives. One of them consisted of oscillations between 30 and 50 °C to study the melting and crystallization, while the other reached temperatures of 260 °C. The latest aimed at stimulating the extrusion conditions, where the capsules are incorporated into the PA6. Each sample was analyzed at least three times for reproducibility purposes. The latent heat of fusion of MF/n-eicosane MCs was calculated with the data obtained by DSC from Equation (2):

(2) $$\Delta H_{f}=\frac{E\left(mJ\right)}{m\left(mg\right)}$$

where *E* (*mJ*) is obtained from the area under the curve in the DSC diagram (power (mW) vs. time (s)) and *m* (*mg*) is the mass of the sample analyzed.

The percentage of encapsulated n-eicosane was calculated from the fusion enthalpies obtained from the DSC curve, as well as from the weight losses obtained by TGA using Equations (3) and (4), respectively:

(3) $$\%\;eicosane_{encapsulated}=\frac{\Delta H_{f}\;\left(MC\right)}{\Delta H_{f}\;\left(pure\;eicosan\right)}\cdot 100$$

(4) $$\%\;eicosane_{encapsulated}=\frac{eicosane\;mass}{MC\;mass}\cdot 100$$

The chemical characteristics of the MCs were studied by FTIR (Bruker’s VERTEX 70v FT-IR Spectrometer, Billerica, MA). The spectrum was obtained in absorbance mode collecting data in the range of 600–4000 cm^−1^.

The n-eicosane retention studies were carried out by solid–liquid extractions in deionized water for 24 h at 60 °C and deionized water with detergent at 60 °C for 2 h and 72 h. After extractions, samples were analyzed by DSC and TGA using the methods previously described.

## 3. Results and Discussion

### 3.1. Characterization of the Microcapsules

The MCs prepared were characterized by several techniques: SEM to check their morphology and size; TGA and FTIR, to corroborate the presence of n-eicosane, and DSC, to calculate the latent heat during the phase transition between liquid and solid states of n-eicosane.

**Figure 2:**
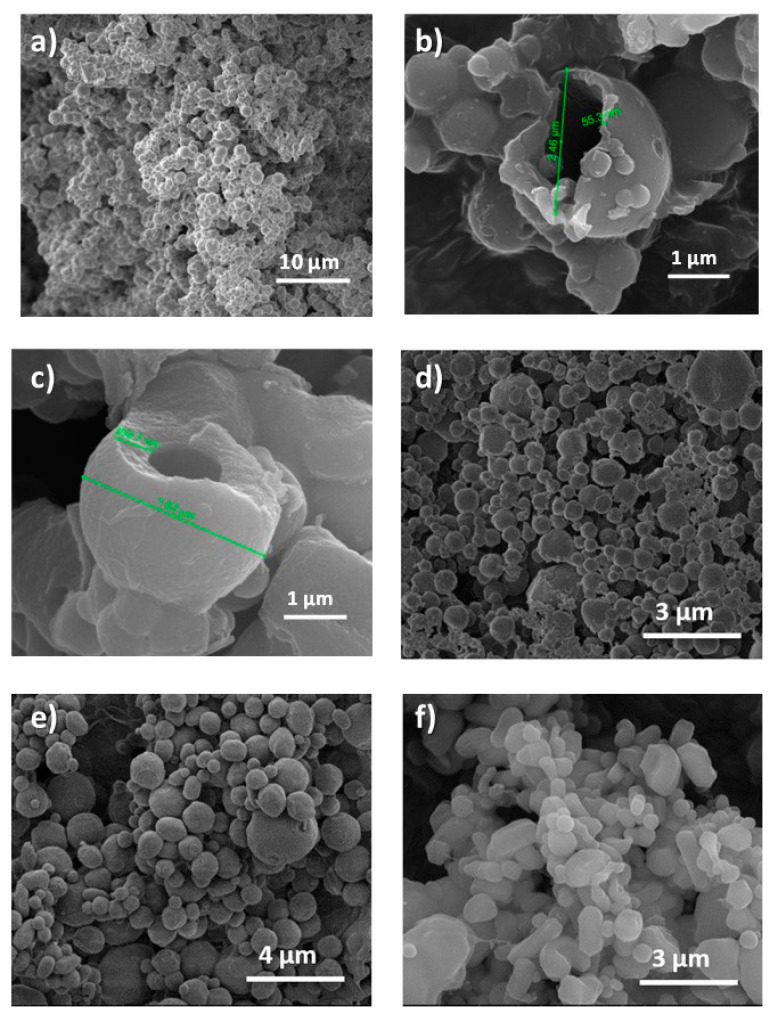
SEM images of MF/n-eicosane microcapsules (**a**) (MC1); (**b**,**c**) individual MF/n-eicosane MC with different wall thicknesses (**d**) MC4; (**e**) MC8; (**f**) MC12.

The SEM images showed that the MCs have a spherical morphology with an average diameter of 1.1 ± 0.4 μm (Figure 2a). Their particle size is in the order of that of Mohaddes et al. [9], which ranged between 0.5 and 2.7 µm. In the literature, using similar procedures, other MF capsules loaded with PCM can be found with different particle sizes (Table 2). Besides, when encapsulating an essential oil, Lee et al. [16] also obtained particles with a similar size (average size of 1.6 µm).

Figure 2b,c show two broken particles from MC1 (see Table 1) when frozen with liquid nitrogen and ground in a mortar. According to these images the polymer forms a shell around the encapsulated material [29]. However, the wall thickness does not appear to be uniform in the two particles. While the MCs in Figure 2b had a wall thickness of 55 nm, the one in Figure 2c had a thicker wall (of 310 nm in size). The reason for this difference in the wall thickness may be related to the procedure used to add the pre-polymer during its preparation.

**Figure 3:**
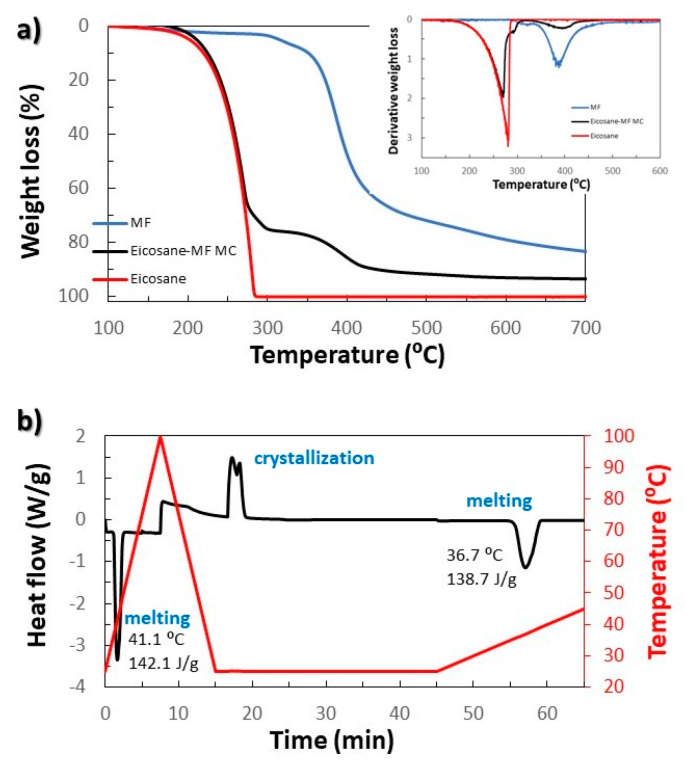
Analysis of the MF/n-eicosane microcapsules (MC1): (**a**) TGA and its 1st derivative; (**b**) DSC.

The thermal stability of the MCs was studied by TGA and derivative thermogravimetric (DTG) analyses. As can be observed in Figure 3a, at 260 °C there is a first mass loss of 71.9 wt.% of the sample which corresponds to the boiling temperature of the n-eicosane. After this first drop, two subsequent losses of 1.9 wt.% and 21.3 wt.% are observed at 311 °C and 383 °C, respectively, corresponding to the degradation of the MF [30].

The melting and crystallization temperatures of the encapsulated n-eicosane were studied by DSC. The n-eicosane encapsulated in the MC1 sample shows a single melting peak (at 36.7 °C if the heating rate is at 1 °C/min and at 41.1 °C when it is at 10 °C/min) but two crystallization peaks (Figure 3b and Figure S2). These two peaks could be due to the subcooling process (also called supercooling) [31]. This process has been observed in microencapsulated samples or emulsions and even for materials that macroscopically do not show this process. In this case, the material begins to solidify below its melting temperature. Furthermore, the n-eicosane can crystallize in two phases: a metastable phase and a most stable crystalline state, which occurs from the metastable phase state on cooling [32].

The latent heat of fusion of the MCs from 4 syntheses, 146.2 ± 5.3 J/g, was calculated from the DSC curves, as already explained (see the example of one synthesis in Figure 3b). Compared to the latent heat of 3 samples of pure n-eicosane (235.2 ± 17.4 J/g), the percentage of n-eicosane contained in the microcapsules is 62.2 ± 6.8 wt.%, a value that is consistent with that obtained by TGA.

As it can be observed in Table 2, other MCs of MF reported in the literature had a latent heat of fusion and a PCM loading within the same order.

**Figure 4:**
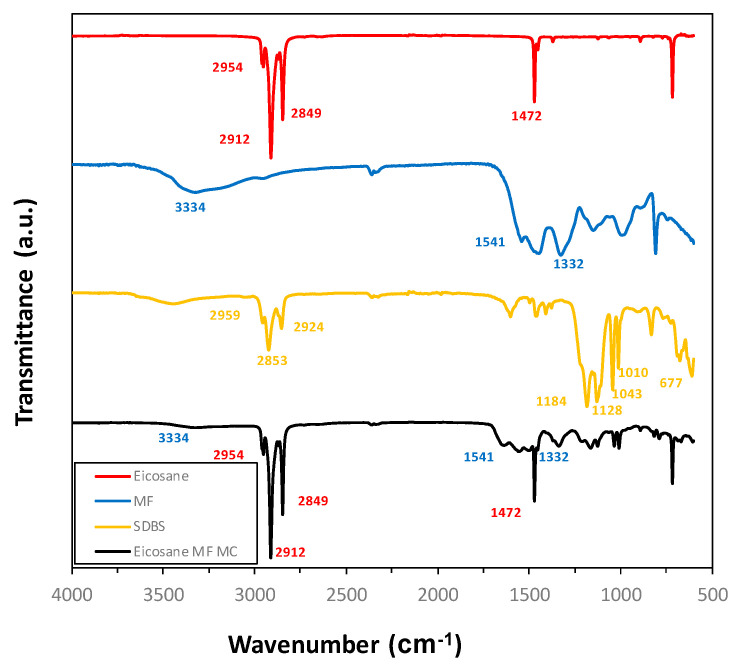
FTIR spectra of n-eicosane, MF polymer, SDBS surfactant and MF/n-eicosane microcapsules MC1.

FTIR spectra are shown in Figure 4. The bands at 3334 cm^−1^, 1541 cm^−1^ and 1332 cm^−1^ correspond to the N-H stretching vibration, the out-of-plane bending vibration of the triazine ring, and the C-N bonding vibration, respectively, all of them being characteristic bands of the MF polymer [9]. Bands corresponding to the surfactant SDBS are also present in the figure, such as the S=O bonding vibrations present at 1184 cm^−1^ and 1043 cm^−1^, the aromatic ring out of plane deformation vibrations at 1128 cm^−1^ and 1010 cm^−1^ and the S-O stretching vibration at 677 cm^−1^. Finally, it can be clearly identified all the bands belonging to the paraffin: at 2954 cm^−1^, 2912 cm^−1^ and 2849 cm^−1^, corresponding to the alkyl C-H bonding vibrations, and at 1472 cm^−1^ and 716 cm^−1^ the deformation vibrations of the methylene groups, which confirm the presence of n-eicosane in the MCs.

### 3.2. Laboratory Scale Process for Microcapsules

To go deeper into the synthesis of the MCs at an industrial scale, it is necessary to previously scale up the process at the laboratory level to study how scaling affects the characteristics of the MCs and the viability of the process. The MCs obtained in the scaling process were observed by SEM and their size and sphericity were studied.

**Figure 5:**
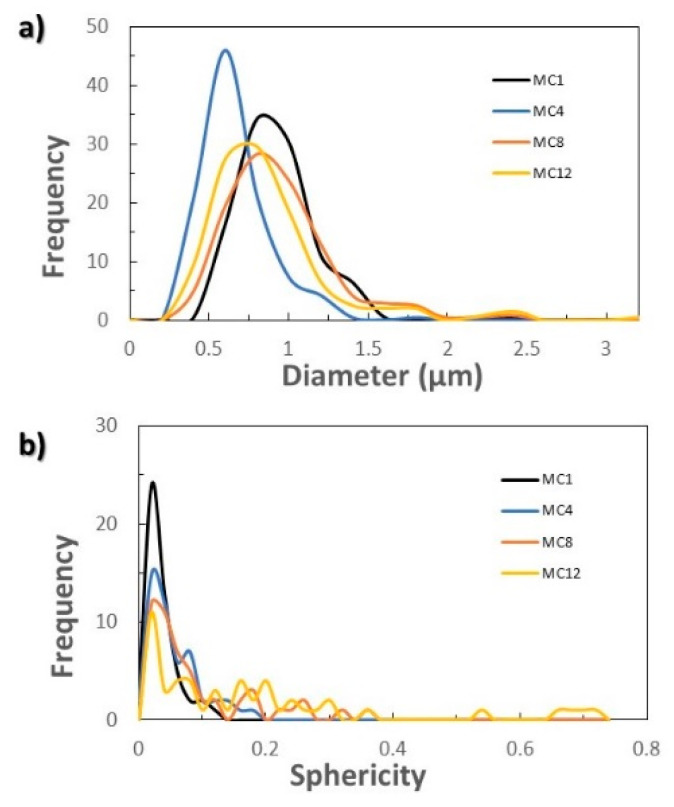
Influence of scaling: (**a**) size distribution of MCs in number; (**b**) distribution of the sphericity factor of the microcapsules in number.

The homogeneity in size, as well as the sphericity, slightly worsened when scaling the process (Figure 2 and Figure 5). In all cases, the majority of MCs had a diameter of 1 μm (Figure 5a). However, a fraction of larger MCs could also be appreciated, reaching up to 3 μm when scaling by 4, 8 and 12 (Figure 2d–f). The sphericity of the capsules progressively decreased as the scaling of the process increased, finding a greater number of capsules with higher sphericity factor as can be observed in Figure 5b (MCM1: 0.04 ± 0.02, MCM4: 0.06 ± 0.04, MCM8: 0.09 ± 0.08 and MCM12: 0.18 ± 0.19). It should be noted that a zero value of the sphericity factor indicates a perfect sphere. For all the samples, the amount of encapsulated n-eicosane calculated with the thermograms of Figure S1 (see the Supplementary Materials file) was in the same range of values, from 67% (MCM12) to 72% (MCM1). The latent heat of fusion of the MCs is maintained by scaling, being 146.9 ± 6.5 J/g calculated by DSC from three syntheses of MCM12.

### 3.3. Stability of MCs with Temperature and Washing Conditions

**Figure 6:**
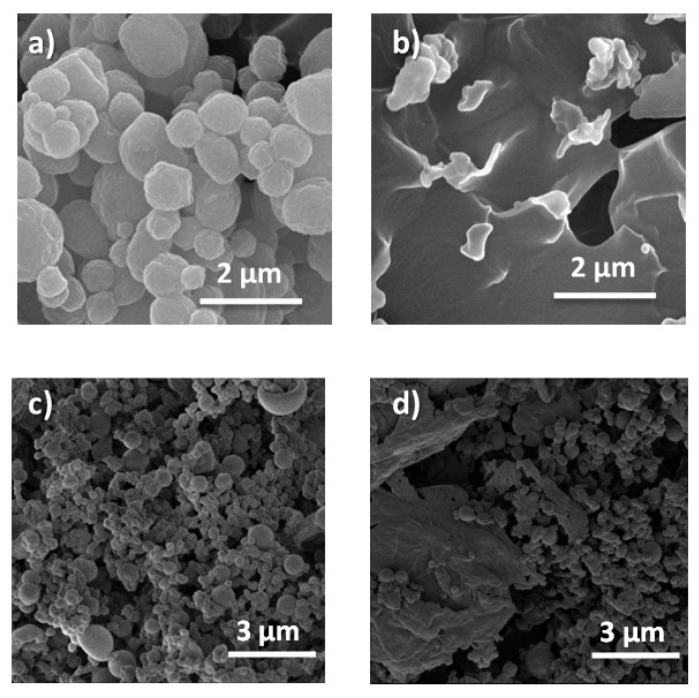
SEM images of MF/n-eicosane microcapsules MC4: (**a**) after half an hour at 260 °C; (**b**) after half an hour at 300 °C; (**c**) after washing with soap and water for 2 h at 60 °C; (**d**) after washing with soap and water for 72 h at 60 °C.

To check the thermal stability of the MCs and ensure that they will resist the temperature conditions during the extrusion process, one sample of MCs was heated at 260 °C, and another at 300 °C, maintaining that temperature for half an hour. Later they were observed by SEM. The microcapsules resist the temperature of 260 °C while they do not withstand 300 °C (Figure 6a,b). MF resin is a thermosetting polymer that begins to decompose at 311 °C (as previously seen in the thermogram of Figure 3). For this reason, the absence of MCs from 300 °C is not expected to be produced because of the decomposition of the polymer, but due to the decomposition or boiling of the n-eicosane that produces an increase in the pressure inside the MCs. Such a high pressure causes the MCs rupture. However, even though the first maximum mass loss of MF/n-eicosane MCs is at ca. 270 °C, over the extrusion processing conditions, the encapsulated MCs start to degrade at lower temperatures (ca. 200 °C, as advised in Figure 3a).

**Figure 7:**
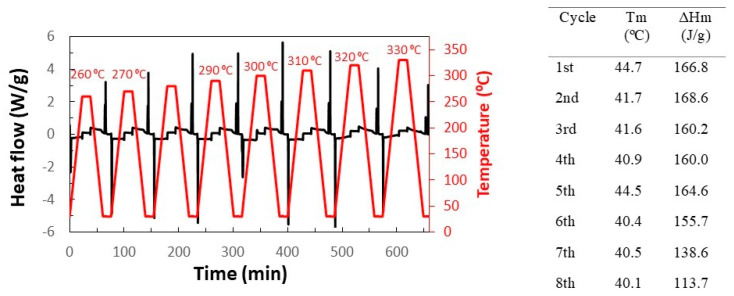
Heating–cooling cycles of the MF/n-eicosane MCs.

SEM images also saw that the aspect of MCs did not vary when they were subjected to 260 °C. However, it is still needed to check that the n-eicosane remains in the capsules. For this purpose, by using DSC, a sample of MCs was subjected to a series of heating-cooling cycles (see Figure 7). The first cycle was heated up to 260 °C, and each subsequent cycle was heated to 10 °C more than the previous one, up to 330 °C. It can also be seen that the latent heat of fusion does not decrease until it exceeds 300 °C, which corresponds to cycle 5.

As explained before, the main purpose of these MCs is their use in the preparation of polymer films as an approximation to textile fibers. Therefore, it is important to analyze the modified materials during their life cycle. To study the performance of the microencapsulated n-eicosane throughout the washing cycles, several extractions were carried out by washing with water and with water and detergent at different temperatures. The thermograms of these experiments are shown in Figure S2a. In all of them, it has been verified that the loss of n-eicosane from the MCs is minimal and that the encapsulated paraffin is not largely lost during the washing process.

DSC experiments showed remarkable differences. When the extraction time is longer, phase changes delay their temperature (Figure S2b). This is because some of the MCs are aggregated during washing, hindering the transmission of heat and delaying the phase transition. The SEM images after the extractions are shown in Figure 6c,d. It can be seen that when the washing lasts 72 h, aggregates of 10–20 μm appear, consistent with the delay in the phase changes seen by DSC. These aggregates were not present in the initial sample.

### 3.4. Properties of Films

For the preparation of the films, the MCM12 capsules were used due to the larger amount of capsules synthesized.

Initially, during the extrusion process, two drawbacks were observed: (*i*) the presence of humidity in the samples, and (*ii*) the size of the agglomerates present in the product that did not disaggregate inside the extruder. To solve these problems a capsule drying step and a micronization stage were added to the procedure, similar to the process followed with zeolite Y capsules when they were used to fabricate functional textile fibers [7]. In addition, other variables such as temperature and pressure were also modified.

The films obtained this way were characterized using various techniques. SEM was used to verify the distribution of the MCs within the polymer matrix, the microcapsule-polymer interaction, and the absence of defects in the films. For that, different areas were taken to observe its surface and cross-section by immersion in liquid nitrogen to favor a clean cut of the section. DSC analysis was used to calculate the heat storage capacities of the films and, therefore, to quantify the n-eicosane additive. Finally, TGA analysis was used to estimate the percentage of PCM in the films. Two different extrusions at 235 °C and 90 bar were made to find the most suitable dispersion of the MCs: one with a 9 wt.% of MCs and another with a 17 wt.%. Regarding the film with 17 wt.% loadings (see Figure S3 in the Supplementary Materials file), larger uneven areas with imperfections and pores were found. These imperfections were possibly created by an excess of MC aggregates, which could be probably formed by the increase in tension during the sample preparation for SEM observation. The 9 wt.% loading film (Figure S4 in the Supplementary Materials file) presented a good visual appearance. By means of SEM some of the MCs were observed inside the film, but to a lesser extent than expected. This film, with three DSC analyses, showed heat storage capacities of 2.3 ± 0.4 J/g, which were far from the 13 J/g that was expected. The high pressure reached during the extrusion (90 bar) could favor some breakdown of the MCs, releasing the n-eicosane. This break would be greater in the MCs with lower wall thickness and, therefore, with higher PCM loading. During the extrusion, a slight smoke was appreciated possibly due to this loss of the n-eicosane. These results are in agreement with Fredi et al. [33], who observed the breaking of MCs and the PCM leaks in the melt compounding of MF MCs with polyamide 12 at 200 °C. Using similar temperatures (180–230 °C) but different polymer (isotactic polypropylene), Salaün et al. [34] prepared melt compounded MF MCs loaded with flame retardant agents. In this case, they observed that the capsules maintained their integrity although, depending on the conditions, aggregates were formed.

**Figure 8:**
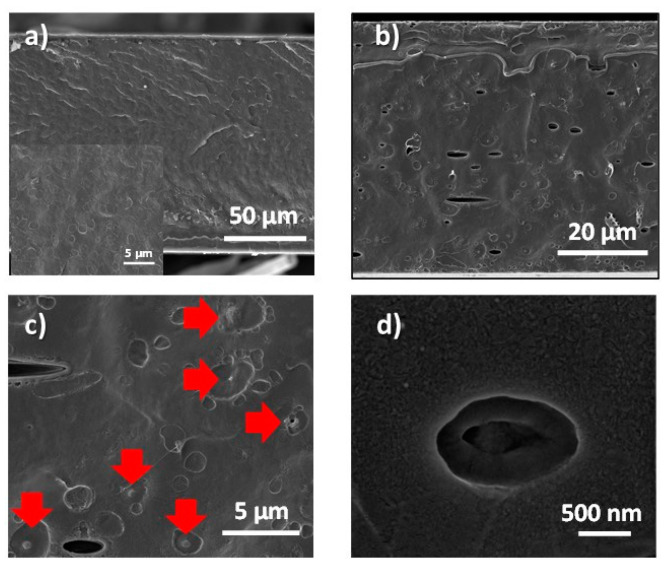
Cross section view of PA6 film made at 256 °C and 50 bar: (**a**) without MCs; (**b**–**d**) with 10 wt.% MF/n-eicosane MCs.

Therefore, to reduce the extrusion pressure, the temperature needs to be increased to 256 °C. In this case, the pressure reached was 50 bar. In view of the previous results, 10 wt.% of MCs were added. Circular marks corresponding to the PA6 polymer were observed by SEM (Figure 8) in both films, with and without MCs. However, the presence of MCs was visible in the film (Figure 8c,d). On the top view of both films, with and without MCs, there are no appreciable differences (Figure S5 in the Supplementary Materials file).

It could also be seen the good interaction of the MCs with the PA6. They did not present interstitial voids and the MCs were well dispersed without noticeable aggregates. This result is in agreement with Fredi et al. [33], where good adhesion between the PA6 and the MF MCs with PCM was observed. As in previous MC film samples, some voids are observed (Figure 8b) that could affect the mechanical properties. In Figure 8d, it can be seen some capsules that show a core-shell structure. DSC analyses of various parts of the film (six areas) revealed similar heat storage capacities, about five times lower than expected (2.6 ± 0.7 J/g). This result can be explained if we take into account the temperature used to prepare the film, which was very close to the one the MCs may withstand. Moreover, it can be due to the loss of n-eicosane during the extrusion process. The value obtained in this work is comparable to that of 2.15 J/g reached by Shin et al. [19] with fabrics that contained 11% MF/n-eicosane capsules. It should be noted that in that study the MCs were impregnated in the fibers, which differs from our strategy, where the MCs have been incorporated into the films during the extrusion process, with the expected advantages in terms of their durability.

**Figure 9:**
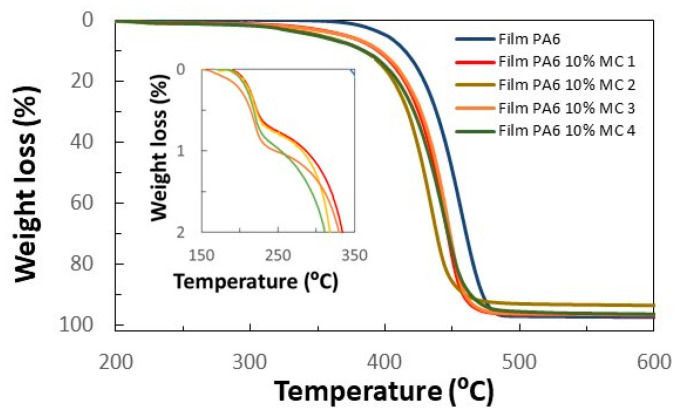
TGA of PA6 film made at 256 °C and 50 bar without MCs and four samples with 10 wt.% MF/n-eicosane MCs. Four different areas of the membrane were considered (MC 1 to MC 4).

TGA analysis of four areas of the film containing 10 wt.% of MCs has additionally been carried out (Figure 9). The TGA curves show a first weight loss between 200 and 250 °C, which is related to the n-eicosane. The subsequent weight loss is related to the degradation of the polyamide occurring at a slightly lower temperature than in the film without MCs. The percentage of PCM can be estimated with the weight loss on the n-eicosane (in this case it was 0.89 ± 0.13% by weight). With this value, and considering the latent heat of the n-eicosane, the heat storage capacity of the films can also be calculated. Therefore, the heat storage capacity of the prepared films was calculated to be 2.09 ± −0.30 J/g, which is very close to that estimated by DSC.

The stability of the MCs was studied with an LDPE film at 133 °C and 181 bar, also with 10 wt.% MF/n-eicosane MCs. This effect was observed by SEM in Figure S6, seeing that the presence of MCs was smaller than expected for 10 wt.% of loading. DSC analyses were performed for five different areas of the film. Fairly high heterogeneity in the latent heat was observed, obtaining an average value of 3.1 ± 3.8 J/g. It suggests that with the high pressure reached (of 181 bar) the n-eicosane would have left preferentially the MCs and be heterogeneously dispersed in the film.

In any case, the process and the MF MCs must still be optimized to avoid the loss of part of the n-eicosane during the extrusion process. In the literature, different materials such as cellulose nanofibers [35] or graphene oxide [34] were added to the MF shell to improve its properties and to extend its current applications.

## 4. Conclusions

In this work, 1 μm diameter MFMCs with 62.2 ± 6.8 wt.% n-eicosane and latent heat of fusion of 146.2 ± 5.3 J/g, calculated by DSC, have been obtained. They have been chemically, thermally, and morphologically characterized by several techniques (including TGA, DSC, FTIR, or SEM) and the process has been scaled up at a laboratory scale. The MCs maintained their appearance and latent heat at 260 °C. In addition, they have been washed with soap and water at 60 °C for 72 h without appreciating the loss of n-eicosane. During scaling, the sphericity of the MCs decreased slightly, and the size distribution of MCs became more heterogeneous, maintaining the average size but forming some capsules of larger size, reaching up to 3 μm.

MF/n-eicosane MCs have been incorporated into PA6 and LDPE films, showing high compatibility between them. PA6 film obtained at 256 °C and 50 bar with 10 wt.% MF/n-eicosane MCs revealed an estimated phase change material percentage of 0.89 ± 0.13% by weight and a heat storage capacity of 2.6 ± 0.7 J/g. These results, however, indicate the need to improve the MF/n-eicosane MCs to withstand the high-temperature conditions and the high pressure to which the capsules are subjected in the extrusion process. This work opens the possibility of using MCs with PCM in extrusion processes, even though the comparison in terms of durability and heat storage between processes, such as the one proposed here and surface treatments, should be explored in more detail.

## Figures and Tables

**Table 1:** Amounts of reagents used in the scaling process.

Name	Melamine (g)	37% Formaldehyde (g)	SDBS (g)	n-Eicosane (g)
MC1	0.6	1.2	1.0	7.5
MC4	2.4	4.8	4.0	30.0
MC8	4.8	9.6	8.0	60.0
MC12	7.2	14.4	12.0	90.0

**Table 2:** Properties of melamine-formaldehyde microcapsules with PCM.

PCM	Particle Size (µm)	Latent Heat of Melting (J/g)	PCM Load (wt%)	Reference
n-octadecane	0.9–9.2	170	70	[8]
n-octadecane	2.2	144	59	[16]
n-eicosane	0.5–2.7	162	75	[9]
n-eicosane	0.1–10	134	53	[19]
paraffin wax	0.26–0.45	107–135	60–74	[17]
1-dodecanol	0.49	79	41	[18]
n-eicosane	1.1 ± 0.4	146.2	62.2	This study

## Supplementary Materials

The following are available online at https://www.mdpi.com/article/10.3390/membranes12030266/s1, Figure S1: TGA of the MF/n-eicosane microcapsules synthesized during the scaling process, Figure S2: Microcapsules MC1 washed in different conditions: (a) TGA; (b) DSC, Figure S3: Cross-section view of polyamide 6 films made at 235 °C and 90 bar with 17 wt.% MF/n-eicosane MCs, Figure S4: Cross-section view of polyamide 6 films made at 235 °C and 90 bar with 9 wt.% MF/n-eicosane MCs, Figure S5: Polyamide 6 film made at 256 °C and 50 bar: (a) surface view without MCs, (b) surface view with 10 wt.% MF/n-eicosane MCs, and (c) cross-section view with 10 wt.% MF/n-eicosane MCs, Figure S6: Cross section view of low density polyethylene film made at 133 °C and 181 bar with 10 wt.% MF/n-eicosane MCs.

## References

[1] Nelson G. (2002). Application of microencapsulation in textiles. Int. J. Pharm..

[2] Valdes A., Ramos M., Beltran A., Garrigos M.C. (2018). Recent Trends in Microencapsulation for Smart and Active Innovative Textile Products. Curr. Org. Chem..

[3] Algieri C., Chakraborty S., Pal U. (2021). Efficacy of phase inversion technique for polymeric membrane fabrication. J. Phase Chang. Mater..

[4] Sherwin C., Bhat S., Hebbar S.P. (2021). Effect of plating time on surface morphology and coating thickness of nickel plating on copper surface. J. Phase Chang. Mater..

[5] Paseta L., Antoran D., Coronas J., Tellez C. (2019). 110th Anniversary: Polyamide/Metal-Organic Framework Bilayered Thin Film Composite Membranes for the Removal of Pharmaceutical Compounds from Water. Ind. Eng. Chem. Res..

[6] Rubio C., Piera E., Angel Caballero M., Tellez C., Coronas J. (2015). Synthesis of layered titanosilicate JDF-L1 for fabrication of composite polyamide 6 film. Appl. Clay Sci..

[7] Perez E., Martin L., Rubio C., Urieta J.S., Piera E., Caballero M.A., Tellez C., Coronas J. (2010). Encapsulation of alpha-tocopheryl acetate into zeolite Y for textile application. Ind. Eng. Chem. Res..

[8] Zhang X.X., Fan Y.F., Tao X.M., Yick K.L. (2004). Fabrication and properties of microcapsules and nanocapsules containing n-octadecane. Mater. Chem. Phys..

[9] Mohaddes F., Islam S., Shanks R., Fergusson M., Wang L., Padhye R. (2014). Modification and evaluation of thermal properties of melamine-formaldehyde/n-eicosane microcapsules for thermo-regulation applications. Appl. Therm. Eng..

[10] Hu X., Huang Z., Zhang Y. (2014). Preparation of CMC-modified melamine resin spherical nano-phase change energy storage materials. Carbohydr. Polym..

[11] Moreira A.C.G., Manrique Y.A., Martins I.M., Fernandes I.P., Rodrigues A.E., Lopes J.C.B., Dias M.M. (2020). Continuous Production of Melamine-Formaldehyde Microcapsules Using a Mesostructured Reactor. Ind. Eng. Chem. Res..

[12] Bône S., Vautrin C., Barbesant V., Truchon S., Harrison I., Geffroy C. (2011). Microencapsulated fragrances in melamine formaldehyde resins. Chimia.

[13] Mercade-Prieto R., Pan X.M., Fernandez-Gonzalez A., Zhang Z.B., Bakalis S. (2012). Quantification of Microcapsules Deposited in Cotton Fabrics before and after Abrasion Using Fluorescence Microscopy. Ind. Eng. Chem. Res..

[14] Chen Z., Wang J., Yu F., Zhang Z., Gao X. (2015). Preparation and properties of graphene oxide-modified poly(melamine-formaldehyde) microcapsules containing phase change material n-dodecanol for thermal energy storage. J. Mater. Chem..

[15] Wang H.P., Yuan Y.C., Rong M.Z., Zhang M.Q. (2009). Microencapsulation of styrene with melamine-formaldehyde resin. Colloid Polym. Sci..

[16] Li W., Zhang X.-X., Wang X.-C., Niu J.-J. (2007). Preparation and characterization of microencapsulated phase change material with low remnant formaldehyde content. Mater. Chem. Phys..

[17] Zhang N., Yuan Y. (2020). Synthesis and thermal properties of nanoencapsulation of paraffin as phase change material for latent heat thermal energy storage. Energy Built Environ..

[18] Kumar G.N., Al-Aifan B., Parameshwaran R., Ram V.V. (2021). Facile synthesis of microencapsulated 1-dodecanol/melamine-formaldehyde phase change material using in-situ polymerization for thermal energy storage. Colloids Surf. Physicochem. Eng. Asp..

[19] Shin Y., Yoo D.-I., Son K. (2005). Development of thermoregulating textile materials with microencapsulated phase change materials (PCM). II. Preparation and application of PCM microcapsules. J. Appl. Polym. Sci..

[20] Onder E., Sarier N., Cimen E. (2008). Encapsulation of phase change materials by complex coacervation to improve thermal performances of woven fabrics. Thermochim. Acta.

[21] Sarier N., Onder E. (2012). Organic phase change materials and their textile applications: An overview. Thermochim. Acta.

[22] Petrosino F., Wickramasinghe S.R., Pal U. (2021). Computational modeling in studying phase change materials. J. Phase Chang. Mater..

[23] Pal U., Chakraborty S. (2021). Towards growth and sustainable researches in phase change materials. J. Phase Chang. Mater..

[24] Okano M., Ogata Y. (1952). Kinetics of the condensation of melamine with formaldehyde. J. Am. Chem. Soc..

[25] Mijatovic J., Binder W.H., Kubel F., Kantner W. (2002). Studies on the stability of MF resin solutions: Investigations on network formation. Macromol. Symp..

[26] Merline D.J., Vukusic S., Abdala A.A. (2013). Melamine formaldehyde: Curing studies and reaction mechanism. Polym. J..

[27] Chan E.-S., Wong S.-L., Lee P.-P., Lee J.-S., Ti T.B., Zhang Z., Poncelet D., Ravindra P., Phan S.-H., Yim Z.-H. (2011). Effects of starch filler on the physical properties of lyophilized calcium–alginate beads and the viability of encapsulated cells. Carbohydr. Polym..

[28] Chotiko A., Sathivel S. (2016). Three protective agents for pectin-rice bran capsules for encapsulating Lactobacillus plantarum. Food Biosci..

[29] Daiguji H., Makuta T., Kinoshita H., Oyabu T., Takemura F. (2007). Fabrication of Hollow Melamine−Formaldehyde Microcapsules from Microbubble Templates. J. Phys. Chem..

[30] Lee A.R., Han C.H., Yi E. (2014). Preparation and characterization of melamine-formaldehyde microcapsules containing Citrus unshiu essential oil. Fibers Polym..

[31] Solomon G.R., Karthikeyan S., Velraj R. (2013). Sub cooling of PCM due to various effects during solidification in a vertical concentric tube thermal storage unit. Appl. Therm. Eng..

[32] Genovese A., Amarasinghe G., Glewis M., Mainwaring D., Shanks R.A. (2006). Crystallisation, melting, recrystallisation and polymorphism of n-eicosane for application as a phase change material. Thermochim. Acta.

[33] Fredi G.D.A., Pegoretti A. (2018). Multifunctional glass fiber/polyamide composites with thermal energy storage/release capability. Express Polym. Lett..

[34] Salaün F., Lewandowski M., Vroman I., Bedek G., Bourbigot S. (2011). Development and characterisation of flame-retardant fibres from isotactic polypropylene melt-compounded with melamine-formaldehyde microcapsules. Polym. Degrad. Stab..

[35] Wu S., Zhang P., Xu Z., Chen Z., Gao Y. (2020). Preparation of 1-dodecanol microcapsules with cellulose nanofibers-modified melamine-formaldehyde resin as a potential phase change material. Mater. Res. Express.

