# Supplementary material for: Study of Melamine-Formaldehyde/Phase Change Material Microcapsules for the Preparation of Polymer Films by Extrusion
Source: Membranes (Basel). 2022 Feb 25;12(3):266. doi: 10.3390/membranes12030266 (PMC8950258; doi:10.3390/membranes12030266)
Supplement: Supplementary file 1 [file membranes-12-00266-s001.zip › membranes-1547350-supplementary.pdf]

membranes

Supplementary materials

Study of Melamine-Formaldehyde/Phase Change Material Mi-crocapsules for the Preparation of Polymer Films by Extrusion

Sara García-Viñuales [1,2], César Rubio [1,2], Lidia Martínez-Izquierdo [1,2], Beatriz Zornoza [1,2,*], Elena Piera [3], Miguel Ángel Caballero [3] and Carlos Téllez [1,2,*]

[1] Instituto de Nanociencia y Materiales de Aragón (INMA), CSIC-Universidad de Zaragoza, 50009 Zaragoza, Spain; sgarvi@unizar.es (S.G.-V.); cesar.rubio@unizar.es (C.R.); limartinez@unizar.es (L.M.-I.)
[2] Chemical and Environmental Engineering Department, Universidad de Zaragoza, 50018 Zaragoza, Spain
[3] Research and Development Department, Nurel S.A., Ctra. Barcelona km 329, 50016 Zaragoza, Spain; epiera@samca.com (E.P.); acaballero@samca.com (M.A.C.)
* Correspondence: bzornoza@unizar.es (B.Z.); (ctellez@unizar.es) (C.T.)

Abstract: n-Eicosane-melamine formaldehyde microcapsules of an average size of 1.1 μm and latent heat of fusion of 146.2 ± 5.3 J/g have been prepared. They have been characterized by scanning electron microscopy, FTIR spectroscopy, calorimetric techniques, and thermogravimetric analyses. Under processing conditions, the microcapsules apparently preserved their properties, also maintaining their n-eicosane loading and heat storage capacity under washing conditions (water with detergent at 60 °C). The microcapsules synthesis has been scaled up for the fabrication of functional films by extrusion. For that, polymer films containing 10 wt.% of microcapsules were prepared at a pilot plant level. In those films, even though a fraction of the n-eicosane loading was lost during the extrusion process, the microcapsules showed good compatibility within the polyamide. The percentage of PCM in the polyamide 6 films was estimated by TGA, verifying also the heat storage capacity predicted by DSC (2.6 ± 0.7 J/g).

Keywords: n-Eicosane-melamine formaldehyde microcapsules; polyamide 6 films; phase change material; Heat storage

Citation: García-Viñuales, S.; Rubio, C.; Martínez-Izquierdo, L.; Zornoza, B.; Piera, E.; Caballero, M.Á.; Téllez, C. Study of Melamine-Formaldehyde/Phase Change Material Mi-crocapsules for the Preparation of Polymer Films by Extrusion. *Membranes* **2022**, *12*, 266. https://doi.org/10.3390/membranes12030266

Academic Editors: Claudia Ursino and Takeshi Matsuura

Received: 24 December 2021
Accepted: 23 February 2022
Published: 25 February 2022

Publisher's Note: MDPI stays neutral with regard to jurisdictional claims in published maps and institutional affiliations.

Copyright: © 2022 by the authors. Licensee MDPI, Basel, Switzerland. This article is an open access article distributed under the terms and conditions of the Creative Commons Attribution (CC BY) license (https://creativecommons.org/licenses/by/4.0/).

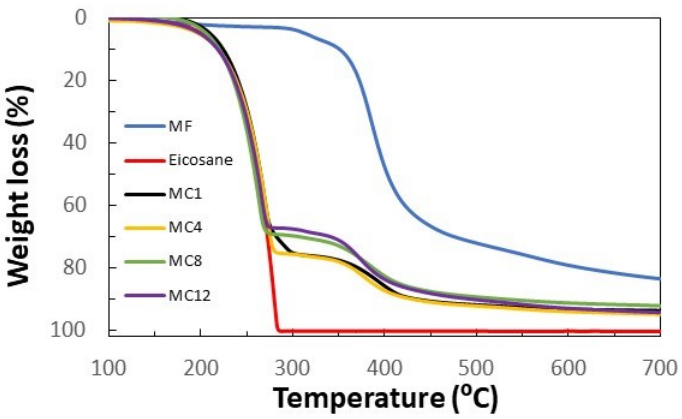

Figure S1. TGA of the MF/n-eicosane MCs synthesized during the scaling process.

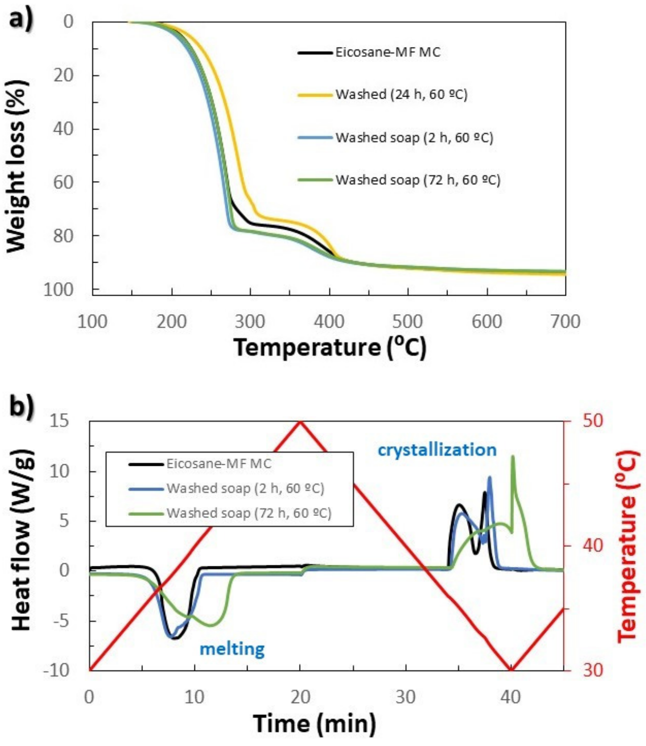

Figure S2. Microcapsules MC1 washed in different conditions: (**a**) TGA; (**b**) DSC.

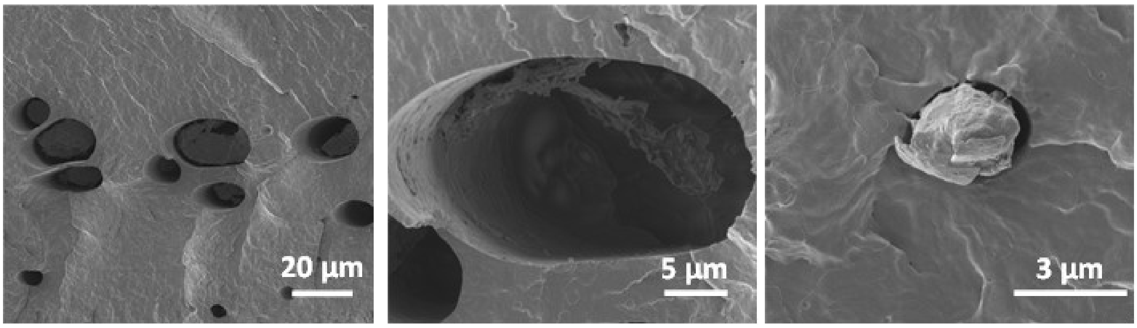

(**a**) (**b**) (**c**)

Figure S3. Cross-section view of PA6 film made at 235 °C and 90 bar with 17 wt.% MF/n-eicosane MCs.

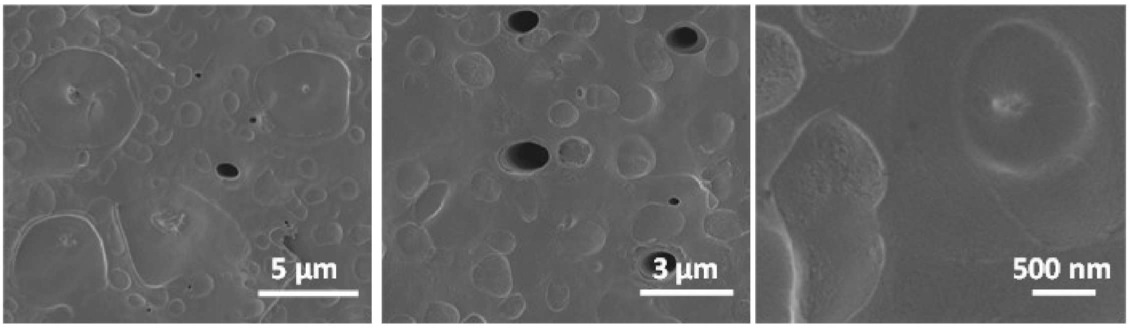

(**a**) (**b**) (**c**)

Figure S4. Cross-section view of PA6 film made at 235 °C and 90 bar with 9 wt.% MF/n-eicosane MCs.

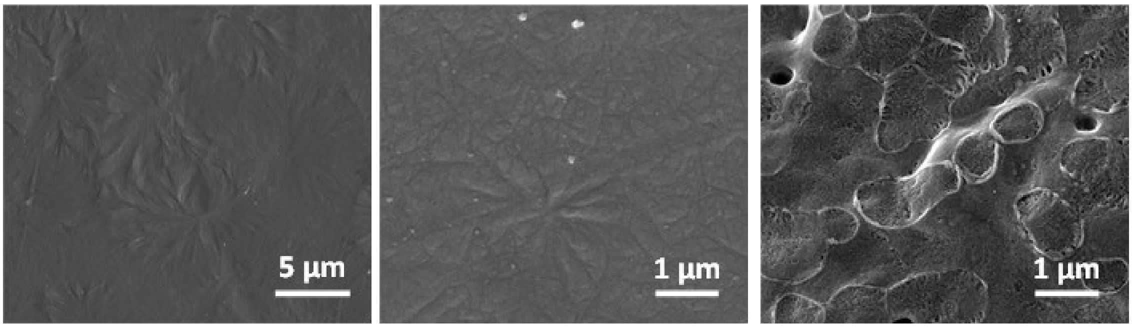

(**a**) (**b**) (**c**)

Figure S5. PA6 film made at 256 °C and 50 bar: (**a**) surface view without MCs; (**b**) surface view with 10 wt.% eicosane-MF MCs and (**c**) cross section view with 10 wt.% MF/n-eicosane MCs.

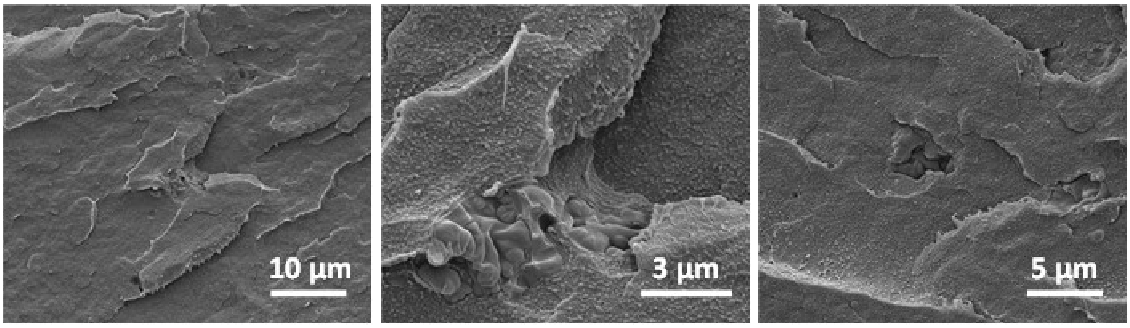

(**a**) (**b**) (**c**)

Figure S6. Cross-section view of low density polyethylene film made at 133 °C and 181 bar with 10 wt.% MF/n-eicosane MCs.
